# Impaired Wound Healing, Fibrosis, and Cancer: The Paradigm of Recessive Dystrophic Epidermolysis Bullosa

**DOI:** 10.3390/ijms22105104

**Published:** 2021-05-12

**Authors:** Grace Tartaglia, Qingqing Cao, Zachary M. Padron, Andrew P. South

**Affiliations:** 1 Department of Dermatology and Cutaneous Biology, Thomas Jefferson University, 233 S. 10th Street, BLSB 406, Philadelphia, PA 19107, USA; grace.tartaglia@students.jefferson.edu (G.T.); Qingqing.Cao@students.jefferson.edu (Q.C.); Zachary.padron@jefferson.edu (Z.M.P.); 2 The Joan and Joel Rosenbloom Research Center for Fibrotic Diseases, Thomas Jefferson University, Philadelphia, PA 19107, USA; 3 Sidney Kimmel Cancer Center, Thomas Jefferson University, Philadelphia, PA 19107, USA

**Keywords:** fibrosis, wound healing, squamous cell carcinoma, recessive dystrophic epidermolysis bullosa, therapies, squamous cell carcinoma

## Abstract

Recessive Dystrophic Epidermolysis Bullosa (RDEB) is a devastating skin blistering disease caused by mutations in the gene encoding type VII collagen (C7), leading to epidermal fragility, trauma-induced blistering, and long term, hard-to-heal wounds. Fibrosis develops rapidly in RDEB skin and contributes to both chronic wounds, which emerge after cycles of repetitive wound and scar formation, and squamous cell carcinoma—the single biggest cause of death in this patient group. The molecular pathways disrupted in a broad spectrum of fibrotic disease are also disrupted in RDEB, and squamous cell carcinomas arising in RDEB are thus far molecularly indistinct from other sub-types of aggressive squamous cell carcinoma (SCC). Collectively these data demonstrate RDEB is a model for understanding the molecular basis of both fibrosis and rapidly developing aggressive cancer. A number of studies have shown that RDEB pathogenesis is driven by a radical change in extracellular matrix (ECM) composition and increased transforming growth factor-beta (TGFβ) signaling that is a direct result of C7 loss-of-function in dermal fibroblasts. However, the exact mechanism of how C7 loss results in extensive fibrosis is unclear, particularly how TGFβ signaling is activated and then sustained through complex networks of cell-cell interaction not limited to the traditional fibrotic protagonist, the dermal fibroblast. Continued study of this rare disease will likely yield paradigms relevant to more common pathologies.

## 1. Introduction

Wound healing is the body’s natural process to restore tissue function and integrity after mechanical or pathological insult. Unresolved wound healing leads to prolonged inflammation, extensive scarring and disrupted tissue architecture, which ultimately results in fibrosis and often leads to organ failure and associated morbidit­ies, including cancer [1]. Fibrosis, characterized by disrupted tissue repair, evolves over time into a progressive and uncontrolled deposition of extracellular matrix (ECM) and fibrosis arguably contributes to the majority of human diseases in one form or another. In the skin, the largest organ of the body (covering the entire external surface), fibrosis can manifest multiple different pathologies such as systematic sclerosis and keloid scars [2,3]. Like all stratifying epithelia, skin is comprised of two major layers: a superficial epidermal layer composed primarily of successive layers of densely packed keratinocytes and a deeper dermal layer consisting of numerous cell types associated with the vasculature, nociceptor nerve endings, hair follicles, as well as the main cell type, the dermal fibroblast [4,5]. Dermal fibroblasts synthesize the majority of ECM components and play a key role in producing and remodeling new ECM upon injury [5]. Under homeostasis, the composition and structure of ECM in normal skin is tightly controlled by the equilibrium between synthesis and degradation over time, and this can be transiently shifted towards ECM synthesis under specific conditions, such as growth and development as well as wound healing [6]. Under pathological conditions, such as in the inherited disease, Recessive Dystrophic Epidermolysis Bullosa (RDEB), disruption to this equilibrium can lead to aberrant deposition of pathological ECM and results in progressive scarring, tissue dysfunction, and altered outside-in signaling, which can ultimately result in cancer [7,8].

## 2. Recessive Dystrophic Epidermolysis Bullosa

RDEB is a rare yet devastating skin-blistering disease caused by loss of function mutations in *COL7A1*, the gene encoding type VII collagen (C7) [9]. RDEB is arguably the most burdensome sub-type of epidermolysis bullosa (EB), a heterogeneous group of skin and mucosal blistering diseases caused by mutations in a wide range of structural proteins important for tissue integrity [10]. Clinical management of RDEB leads to significant healthcare costs throughout the United States [11] and, although multiple clinical trials are underway, and in some cases, completed, RDEB has yet to benefit from an approved therapeutic intervention [12]. RDEB is characterized by digit contraction, healing with scarring, and progressive fibrosis, the net result of which is the development of highly metastatic squamous cell carcinomas (SCC) of the skin—up to 90% of patients with the severe-generalized form of RDEB will succumb to cancer during their lifetime [13].

Historically, lack of epidermal—dermal adhesion was thought to be the primary pathology driving disease severity in RDEB [14]. However, with advances in clinical care and careful research, we have begun to appreciate that the major differences between RDEB and other sub-types of EB (that are not burdened with extensive fibrosis, digit contraction, or risk of developing cancer) are driven primarily by signaling events in dermal fibroblasts [15,16,17,18], although other cell types, such as keratinocytes, have been implicated [18,19,20]. While animal models recapitulated the phenotype in RDEB and identified increased transforming growth factor-beta (TGFβ) signaling [18,21], studies in primary human cells clearly demonstrated that absent expression of wild-type C7 in RDEB dermal fibroblasts or knockdown of C7 in normal dermal fibroblasts leads to increased TGFβ signaling and a disruption to ECM protein organization and composition that is tumor-promoting [15,17]. Since these early studies, fibrosis and tumor development in RDEB have been attributed to substantial disruption to TGFβ signaling [22,23,24,25,26], yet the root cause of aberrant TGFβ signaling remains incompletely characterized. Thus, although TGFβ has emerged as a major disease modifier in RDEB and has provided a unifying mechanism for a range of independent studies identifying diverse routes to TGFβ activation [22,23,24,25,26], data do not identify a single, unifying mechanism of activation and likely points to a combination of contributing factors [22]. In the following table (Table 1), we summarize known molecular targets for RDEB therapeutics and their mechanisms of action.

## 3. Impaired Wound Healing in RDEB

**Figure 1:**
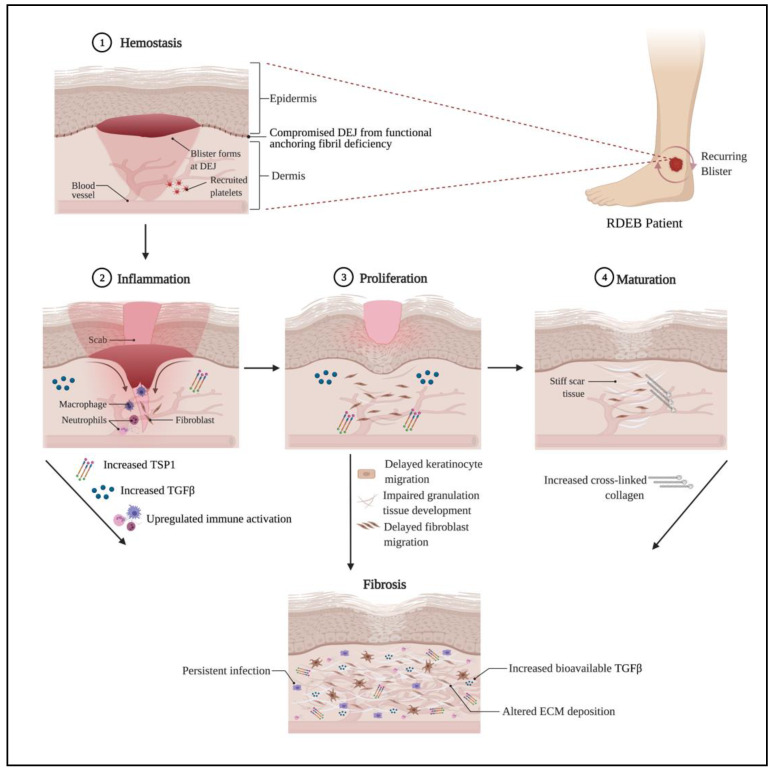
Wound Healing Complications in RDEB. A schematic showing the process of wound healing in its four stages: hemostasis, inflammation, proliferation, and maturation. In RDEB, blister formation triggers the wound healing cascade, which contributes to the increased expression of TGFβ and TSP1, among others. Excessive amounts of these factors contribute to delays in keratinocyte and fibroblast migration, slowing down wound closure. There can also be a persistent infection in the wound area, denoted by the increased presence of activated immune cells. In the event of unresolved maturation, fibrosis occurs, which is demarked by altered ECM deposition. Crosslinked collagen in the wound bed is a prominent component of excessive ECM deposition, leading to the heavy scarring phenotype. Created with Biorender.com.

Arguably, impaired wound healing is the most debilitating pathology in patients with RDEB and is driven by compromised epidermal-dermal adhesion as well as aberrant signaling. In the most comprehensive study of wound healing in an RDEB mouse model to date, major contributions from both the epidermal layer and dermal layer were identified. Delayed migration of keratinocytes was attributed to disorganized laminin-332—integrin a6b4—and subsequent signaling and delayed dermal healing, characterized by abnormal ECM expression, abnormal localization of myofibroblasts, and prolonged presence of inflammatory cells [18]. These observations, made in animal models have been supported by studies of human dermal fibroblasts [15,17] and point to defects arising in the final three of four major stages of wound healing: inflammation, proliferation, and maturation (Figure 1). The initial phase of wound healing, hemostasis, occurs immediately after injury prompting damaged vessels to constrict and minimize blood and lymphatic fluid loss. Recruited platelets block off the break in the blood vessel walls and release TSP-1 through the modulation of the cyclic adenosine monophosphate (cAMP) signaling pathway [27], as well as fibrin threads to reinforce the platelet plug. By downregulating the cAMP pathway, TGFβ is also activated since cAMP has an inhibitory effect on TGFβ’s canonical and non-canonical pathways [28,29,30,31]. The clot serves as a matrix for inflammatory cells to travel to the wound site, release cytokines, such as high mobility group box 1 protein (HMGB1), tumor necrosis factor-alpha (TNFα), interleukin 6 (IL6), and TGFβ, and induce hemostasis [32]. Although not identified in animal models, it is possible that RDEB patients could have complications in hemostasis during the coagulation step if they suffer from severe anemia [33], which is a common pathology (~68% of RDEB patients had anemia in one case series [34]) and manifests from an early age [35] leading to low iron levels which have documented impact on wound healing [36]. Further studies on the role of anemia in hemostasis and later stages of wound healing in RDEB need to be pursued to fully understand the complexities behind potential deficiencies in wound healing since the role of anemia in wound healing itself remains unresolved [36].

Inflammation occurs soon after injury when swelling from the blood vessel exudate accumulates. The fluid promotes healing and guides inflammatory cells, first neutrophils in large numbers followed by macrophages, to the wound site to prevent infection and control bleeding. The inflammatory stage persists through the proliferation stage and resolves before the final stage, maturation, is complete. As such unresolved inflammation is a constant to non-healing wounds and therefore a major focus of the wound healing field. Dogma describes macrophage clearance of short-lived neutrophils as essential for wound resolution, and chronic wounds often exhibit reduced markers of macrophages and increased or persistent evidence of neutrophils [37,38,39,40,41]. Animal models, for the most part, support these concepts yet there are exceptions, and we still have an incomplete understanding of the inflammatory stage of wound healing [42]. Conceptually, numerous barriers to inflammation resolution exist, such as delayed or incomplete re-epithelialization, unresolved microbial infection, persistent damage through epithelial fragility, and presence of cancer or pre-cancerous lesions inducing tumor inflammation and preventing appropriate differentiation and re-epithelialization of normal keratinocytes. RDEB wounds have been shown to exhibit microbial dysbiosis [43,44] and would certainly contribute to delayed healing, while studies of RDEB murine models show that collagen VII plays a role in lymphatic conduits leading to impaired immune response to bacterial colonization [19]. Work analyzing 133 discarded wound dressings from 51 EB patients showed that in chronic wounds (those that failed to heal for 3 months), granulocytes were increased, potentially due to lower numbers of macrophages, and likely contribute to persistent inflammation [38].

Typically, once the wound has formed clots and is filled with inflammatory cells, the extracellular collagen bed will start rebuilding the new tissue during the proliferation stage of wound healing. The collagen-secreting fibroblasts in the fibrin clot differentiate into alpha-smooth muscle actin (α-SMA) expressing myofibroblasts and contribute to contraction to facilitate new tissue construction. Re-epithelialization of proliferating and migrating keratinocytes on the surface occurs and is expedited by hydrated wound beds from occlusive dressings [45]. In the case of RDEB, a brief report of the clinical characteristics of wounds in these patients has noted the association of increased wound sizes with more pain, itchiness, wound closure delay, and high infection risk [46]. In animal models, the delay in wound closure has been attributed to perturbed migration of RDEB keratinocytes as a result of increased activation of the c-Jun N-terminal kinase (JNK) and protein kinase B (AKT) pathways, as well as delayed fibroblast migration and impaired granulation tissue development [18]. In chronic wounds, which is a common feature of RDEB, continuous activation of cells involved in the proliferation step can lead to senescence or depletion. While senescent fibroblasts do have a role in promoting wound closure through growth factor secretion [47], their prolonged presence is also detrimental to skin repair in chronic wound sites [48].

Finally, the maturation step of wound healing is also called the remodeling stage, in which the wound fully closes, all inflammatory cells and myofibroblasts are removed via apoptosis or macrophage clearance, and the ECM is remodeled to resemble homeostatic tissue. The thick collagen matrix bed from the proliferation phase contains cross-linked collagen fibers that contribute to firmer skin over the wounded area, even though the healed skin is still weaker than intact skin [49]. When the maturation stage does not resolve and the ECM remodeling becomes abnormal, the fibrotic reaction continues, and excessive collagen is deposited. Dense scar tissue, due to the elevated amount of collagen fibers, is another distinct feature of RDEB. Long-term damage due to uncontrolled fibrosis includes pseudosyndactyly [50], contributing to a decrease in life quality for these patients [51].

RDEB is unique in being a disease where tissue damage, continued wound healing, bacterial challenge, and fibrosis lead to inevitable and lethal cancers [52]. Animal models of tissue damage-driven cancer identify innate immunity as a potential driver, and a connection between wound healing and tumor formation has been identified in high-mobility group box 1 (HMGB1) [53], a protein derived from keratinocytes that activate inflammation and have been used to induce homing of specific subsets of bone marrow-derived pre-cursors [54]. HMGB1 has also been identified as a highly upregulated protein in RDEB patient tumors [53]. More recent work has identified wound-induced tumors in mice to be dependent on HMGB1 forming neutrophil extracellular traps (NETs), which have antimicrobial peptides that kill pathogens while also damaging tissue [55] and a role for NETs in RDEB may emerge with further investigation.

## 4. TGFβ and Fibrosis in RDEB

TGFβ belongs to a large superfamily of activins and bone morphogenetic proteins that play an important role in the cellular synthesis of ECM proteins, proliferation, and wound healing [56]. Three separate TGFβ isoforms exist, and each is synthesized as a large polypeptide that is cleaved to yield the latency-associated protein (LAP) and the TGFβ ligand [57]. The LAP-TGFβ complex associates non-covalently in the endoplasmic reticulum (ER), is secreted by nearly all cell types and can be stored as a latent complex in the ECM, associating with latent transforming growth factor-beta binding proteins as well as fibronectins and fibrillins [58]. The activation of ECM-bound TGFβ can occur through many different mechanisms, including but not limited to effector proteins binding to the LAP-TGFβ complex, mechanical strain increasing the accessibility of the LAP-TGFβ complex, proteolytic degradation of LAP-TGFβ complex, as well as decreased pH [58]. Non-canonical TGFβ activation can occur through pathways independent of ligand binding, such as mitogen-activated protein (MAP)-kinase or phosphoinositide 3 (PI3)-kinase [59]. For an in-depth review of the TGFβ pathway, please see this citation [60]. Endoplasmic reticulum stress (ERS), the related unfolded protein response, and markers of autophagy have also been implicated in either enhancing or driving fibrosis in numerous model systems, primarily lung [61] but also liver [62] and heart [63]. In each case, there are clear implications for the role of TGFβ signaling, but only a few studies have made a direct link between cellular stress responses and TGFβ [64,65]. The resulting network of TGFβ activating nodes is further complicated by many of the mediators being transcriptional targets of TGFβ itself, leading to feed-forward amplification. Indeed, the matricellular protein TSP1, a well-described activator of TGFβ through LAP binding [66], is itself a TGFβ target [58], and its expression is frequently increased as a result of TGFβ signaling, leading to a “chicken and egg” scenario with speculation over cause or effect. Conceivably, a positive feedback mechanism operates where increased TGFβ signaling upregulates ECM gene expression, leading to both increased activation of latent-TGFβ and potential disruption to proteostasis and increases in ER-stress, the unfolded protein response (UPR), ER-phagy, and other related outcomes that may increase TGFβ signaling [67,68].

In the context of acute injury, TGFβ is released from the ECM through a wide variety of mechanisms, such as platelet secretion of TSP1, mechanical strain, or reactive oxygen species (ROS) generation [58,69]. TGFβ itself can also be secreted by effector cells in the immediate proximity, contributing to the initiation of signaling during the early stages of wound healing [69]. In the later stages of wound healing, TGFβ acts to stimulate wound contraction through triggering α-SMA expression in fibroblasts and inducing myofibroblast differentiation, followed by deposition of a specialized ECM network favoring tissue repair [69]. In this context, while fibroblasts are considered the central effectors converting to myofibroblasts driven by upregulated TGFβ, other cell types have emerged as playing equally significant contributions, such as macrophages and keratinocytes, which are critical to re-epithelialization [70]. In chronic wounds and fibrotic tissues, the TGFβ signaling cascades are constantly activated, leading to excessive production of ECM, immune suppression and inhibition of proliferation [59].

Recent studies have suggested multiple pathological pathways activating TGFβ signaling in RDEB skin. TSP1 has higher bioavailability to activate TGFβ ligands in the ECM of RDEB patients since binding between C7 and TSP1 prevents TSP1 from activating the TGFβ pathway [23]. In addition, inflammation in RDEB skin leads to oxidative imbalance and TGFβ activation, triggering ECM protein synthesis, such as tenascin, periostin and fibronectin 1 [71]. Recent work has shown that RDEB fibroblasts have a greater ability to activate latent TGFβ when added in culture, and this is significantly increased after co-culture with keratinocytes, suggesting a role for both cell types in TGFβ activation in RDEB [22]. Proteomic studies of RDEB fibroblasts and keratinocytes have identified vesicle traffic, autophagy, and lysosomal changes, which potentially contribute to disrupted proteostasis and increase in ER-stress, providing a non-canonical route for TGFβ activation [17,20].

Previous attempts to globally block the TGFβ ligand or receptors for the treatment of other diseases have resulted in developmental defects in vivo, chronic inflammation, and increased cancer risk [72]. Given the essential role of TGFβ in tissue homeostasis, the difficulty lies in finding a modulator of TGFβ that can selectively target abnormal levels without affecting baseline requirements. In RDEB, preclinical efforts have used viral transduction of decorin, an inhibitor of TGFβ, to show a reduction of fibrosis in mouse models [73]. In vitro work has suggested that TSP1 has potential as a therapeutic target for RDEB patients [23], while in vivo work in other disease models shows TSP1 inhibition to have preclinical efficacy [74]. Since diverse mechanisms have been characterized for activating latent TGFβ in RDEB [22], suppressing one latent TGFβ activator may not be sufficient to inhibit fibrosis globally, and the use of inhibitors targeting multiple latent TGFβ activators may need to be considered [75]. One drug that has been shown to inhibit fibrosis in RDEB is losartan, a small-molecule angiotensin II type 1 receptor antagonist, which is commonly used to treat hypertension. Preclinical studies in an RDEB mouse model showed that losartan is capable of significantly lowering levels of TGFβ, thus decreasing inflammation and ECM remodeling [76]. As a result, there is currently a phase I/II clinical trial by the University of Freiburg (EudraCT Number: 2015-003670-32) to determine the safety and efficacy of losartan for improving disease severity in children with RDEB. The link between persistent wounding, fibrosis, and cancer has a long history [77], and while the role of ECM in driving cancer is a newer concept, it has also achieved consensus as a critical mechanism in tumor development [78,79]. Altogether, these various effects of fibrosis contribute to the increased risk of cutaneous cancer in RDEB.

## 5. Cutaneous Cancer in RDEB

SCCs arising in stratifying epithelia (found in numerous anatomical tissues that form a barrier between us and our environment) contribute substantial cancer incidence and mortality yearly, with large numbers in lung cancer as well as esophageal and head neck cancers [80]. Cutaneous SCC (cSCC) arising in the general population are typically associated with ultraviolet radiation exposure and are routinely and effectively treated early with surgical excision. cSCCs are the second most common non-melanoma skin cancer in the United States, but it is difficult to find an exact mortality rate in the general population due to the lack of tracking in the national tumor registry [81]. For patients with RDEB, SCC is common and inevitably aggressive, with a five-year survival rate nearing 0% after initial diagnosis of their first tumor [13].

**Figure 2:**
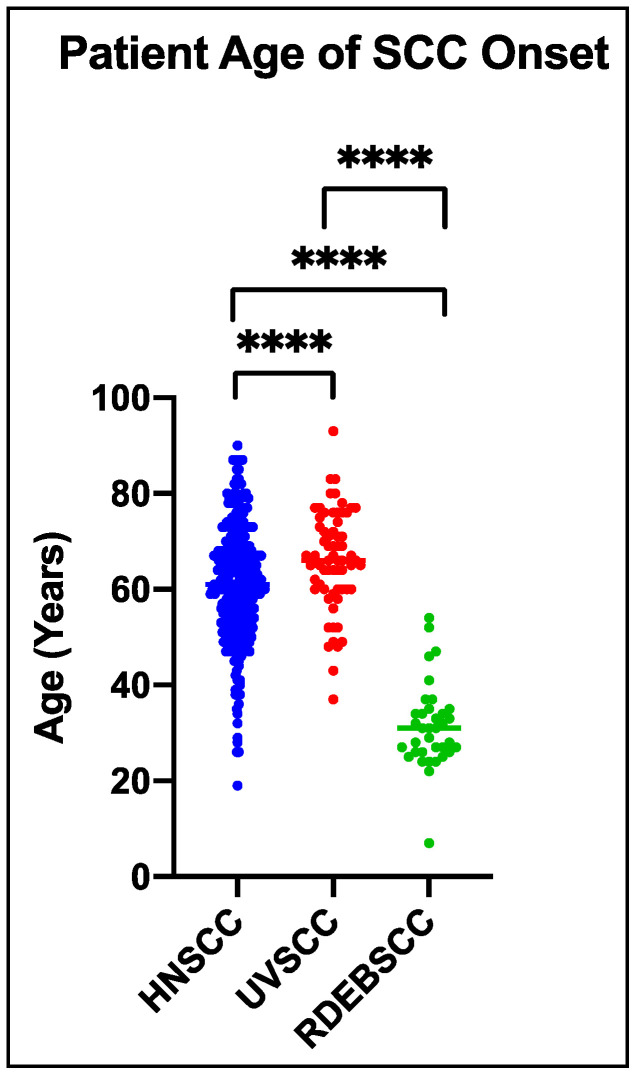
Age of Onset for SCCs in Different Populations. Head and Neck SCC (HNSCC) data [82], UV light-induced SCC (UVSCC) data [83,84], and RDEB SCC (RDEBSCC) data from our records of patient samples accumulated from 2005–2017. Data were plotted by GraphPad-Prism using a two-tailed, Mann–Whitney test assuming unequal variance. ****: *p* ≤0.0001. HNSCC: *n* = 278; UVSCC: *n* = 66; RDEBSCC: *n* = 36.

While the incidence of SCC in RDEB increases with increasing disease severity (a greater chance of developing SCC is observed in patients with the most severe sub-type, severe RDEB, compared with intermediate RDEB), mortality tracks incidence [13], suggesting that the risk of developing SCC in RDEB is dependent on the extent of tissue-damage while the development of metastasis is dependent on RDEB. The risk of SCC development in RDEB is not only high but also accelerated, with the median age of presentation half that of other SCC subtypes (Figure 2). Since C7 is not a classical tumor suppressor (patients heterozygous for *COL7A1* mutation are not at increased risk of developing cancer, and *COL7A1* mutation has not been associated with any non-RDEB cancer to date), the incidence of SCC in RDEB seems not to be driven by cell-intrinsic effects of mutation in *COL7A1* but rather extrinsic effects of lack of C7.

As with all cancer, exceptions to this rule exist, and we are aware of a number of RDEB patients who have had multiple cSCC develop over many years without succumbing to metastatic disease, which in itself speaks to genetic modifiers or specific exposures that are protective or induction of metastasis. However, to date, no unique genetic differences have been identified in RDEB SCC that explain the aggressive nature—all driver gene mutations are shared with other SCC and no genetic element, other than arising on a background of *COL7A1* mutation, has distinguished RDEB SCC from other SCC [85]. One SCC driver mutation that is enriched in RDEB SCC compared with other SCC is *CASP8* [86], a gene in which mutations have been associated with highly immune infiltrated tumors from the pan-cancer analysis [87]. While *CASP8* mutation does not explain the aggressive nature of RDEB SCC, it does point to RDEB SCC being “immune hot,” which has been linked to immune checkpoint success [88] (discussed below) and could be a potential genetic target for future therapy development.

Gene set enrichment analysis comparing the transcriptional change in RDEB SCC with all major sub-types of SCC (including UV induced cSCC, cervical SCC, and lung SCC) identified most similarity with head and neck SCC (HNSCC). This similarity was closest to the mesenchymal and basal sub-types of HNSCC as determined from bulk RNA-sequencing [82,85]. These similarities were confirmed by single-cell sequencing of HNSCC, which identified the mesenchymal subtype of HNSCC is, in fact, the basal subtype with increased stromal contribution from fibroblasts, endothelial, and immune cells [89]. Intriguingly, this study of HNSCC identified the basal/mesenchymal subtype of HNSCC were metastatic while the other major subtypes of HNSCC determined by bulk RNA-sequencing, the classical and atypical HNSCC subtypes, were not metastatic. In basal/mesenchymal HNSCC, tumor metastasis correlated with the presence of tumor cells at the invading edge (the stromal/ tumor interface) that were characterized by a partial-epithelial to mesenchymal transition (p-EMT) transcriptome, driven by activation of TGFβ [90]. Therefore, similarities between RDEB SCC and HNSCC are, in part, driven by activation of TGFβ identified in RDEB SCC fibroblasts [15] and RDEB murine models [21] years earlier. Since the response to TGFβ ligand in RDEB SCC keratinocytes in culture is heterogeneous [91], which is similar to most other cancers studied in the context of TGFβ [92], it is more likely that the primary driving role of TGFβ in SCC is within the tumor microenvironment, affecting cancer-associated fibroblasts, immune cells, and other components, rather than a direct role on the tumor keratinocytes themselves. Since it has been demonstrated that activated wound fibroblasts support tumor development, it is likely that wound fibroblasts contribute to heterogeneous populations of cancer-associated fibroblasts (CAF) in the tumor stroma [93]. However, further research into the complexities of TGFβ signaling in the tumor microenvironment in SCC is needed to fully understand how we might harness this knowledge for effective therapy development [94].

Therapeutic development is still at an early stage, and no approved therapies exist specifically for RDEB SCC. Similarities identified between RDEB SCC and other forms of SCC do support the use of therapies approved for SCC [85]. In this context, the use of both EGFR inhibitors and immune checkpoint inhibitors have been used on individual cases with mixed results [95,96], but no formal clinical trials in RDEB have been conducted to date. One drug being investigated for the treatment of SCC in RDEB patients is rigosertib, an experimental allosteric kinase inhibitor, which was identified initially as a polo-like kinase-1 inhibitor [97]. In vitro and in vivo work using RDEB SCC keratinocyte cultures showed induction of apoptosis in all ten SCC cell lines tested with no adverse effects to primary normal keratinocytes [98]. This work has led to two clinical trials to assess the efficacy and tolerability of this drug in RDEB SCC (NCT03786237, NCT04177498). Other kinase inhibitors have been explored in RDEB. Ruxolitinib, an FDA-approved Jak1/2 inhibitor, has also been tested in vitro on RDEB cSCC, showing successful targeting of STAT3 and successful tumor reduction in vivo [99]. However, in studies of this drug in RDEB mouse models, ruxolitinib showed considerable toxicity [100].

One recent study used unrestricted somatic stem cells (USSCs), thought to be precursors of mesenchymal stem cells derived from human cord blood, to try and suppress fibrosis and malignant progression of SCC in an RDEB animal model [101]. In this study, non-treated mice inherently show an increase in MMP-9 and -13 in the dermis, which are markers for malignant transformation [102,103]. Treatment with USSCs revealed a decrease in MMP and TGFβ signaling, suggesting that USCCs show strong potential in reducing fibrosis formation and SCC formation [101]. Another group has also reported that MMP13 proves to be a successful diagnostic tool for differentiating SCC and benign hyperkeratotic lesions in RDEB as MMP13 appears to have very high expression in SCC lesions [104]. Although this study’s focus is more tailored towards diagnostics, others have taken to the therapeutics route. Ribozymes targeted towards MMP-13 mRNA have shown success in vivo by inhibiting MMP-13 expression and activity in SCC [105]. In the past, MMP inhibitors have shown little to no success in clinical trials due to poor validation of drug targets and lack of specificity. However, current in vitro and in vivo studies have shown higher specificity for targeting MMP-13 but with varying responses based on age [106]. As of yet, none of these novel inhibitors have been tested on RDEB SCC, only in general cSCC cell populations.

## 6. Closing Remarks about Parallels Between RDEB and Aging Pathologies

In the general population, cSCC rates of incidence are increasing, especially in aged populations [107]. In RDEB, tumorigenesis is accelerated and models aggressive disease in aged populations, particularly showing overlap with HNSCC [85]. Poor wound healing arguably affects aging populations with increases in venous leg ulcers and diabetic foot ulcers [5,32], while the same can be said about fibrotic ailments such as pulmonary fibrosis [1]. While fibrosis and poor wound healing are features of RDEB, few studies have compared RDEB and aged skin. One purely transcriptomic study compared RDEB skin, non-RDEB elderly patient skin, and healthy younger patient skin identified a number of potential transcriptomic targets [50]. Further study of RDEB will likely yield mechanistic insight relevant to more common pathologies in the general aging population.

## Figures and Tables

**Table 1:** Potential targets for therapy in RDEB. A brief summary of the key players involved in RDEB pathology and their mechanisms of action.

Potential Target	Underlying Mechanisms
TGFβ	Excessive TGFβ is the main driver of fibrosis via activating myofibroblasts, producing aberrant ECM and inhibiting ECM degradation
Decorin	Decorin potentially reduces TGFβ and fibrosis and is downregulated in RDEB skin
Thrombospondin-1 (TSP1)	TSP1 is an activator of TGFβ pathway and upregulated in RDEB fibroblasts
HMGB1	HMGB1 is upregulated in RDEB, and inhibiting HMGB1 reduces inflammation
STAT3	STAT3 is constitutively activated in RDEB-derived keratinocytes
Matrix Metalloproteinase (MMP)	Increased MMP expression is detected in RDEB skin leading to imbalanced synthesis-degradation in the ECM

## Data Availability

Not applicable.

## Abbreviations

AKT	Protein Kinase B
C7	Type VII Collagen
CAF	Cancer-Associated Fibroblasts
cAMP	Cyclic Adenosine Monophosphate
cSCC	Cutaneous Squamous Cell Carcinoma
EB	Epidermolysis Bullosa
ECM	Extracellular Matrix
ERS	Endoplasmic Reticulum Stress
HMGB1	High Mobility Group Box 1 protein
HNSCC	Head and Neck Squamous Cell Carcinoma
IL6	Interleukin 6
JNK	c-Jun N-terminal Kinase
LAP	Latency Associated Peptide
MAP	Mitogen-Activated Protein
MMP	Matrix Metalloproteinase
NETs	Neutrophil Extracellular Traps
p-EMT	Partial-Epithelial to Mesenchymal Transition
PI3	Phosphoinositide 3
RDEB	Recessive Dystrophic Epidermolysis Bullosa
ROS	Reactive Oxygen Species
SCC	Squamous Cell Carcinoma
STAT3	Signal Transducer and Activator of Transcription 3
TGFβ	Transforming Growth Factor Beta
TNFα	Tumor Necrosis Factor Alpha
TSP-1	Thrombospondin-1
UPR	Unfolded Protein Response
USSCs	Unrestricted Somatic Stem Cells
α-SMA	Alpha-Smooth Muscle Actin
